# Iris recognition approach for identity verification with DWT and multiclass SVM

**DOI:** 10.7717/peerj-cs.919

**Published:** 2022-03-23

**Authors:** Mohamed A. El-Sayed, Mohammed A. Abdel-Latif

**Affiliations:** 1 Technology Department, Applied College, Taif University, Taif, Saudi Arabia; 2 Mathematics Department, Faculty of Science, Fayoum University, Fayoum, Egypt; 3 Mathematics Department, Faculty of Science, South Valley University, Qena, Egypt

**Keywords:** Iris recognition, Dentition, Verification, DWT, SVM, Daugman model, Biometrics feature, Hough transform, Histogram, Iris dataset

## Abstract

The iris has been proven to be one of the most stable and accurate biometrics. It has been widely used in recognition systems to determine the identity of the individual who attempts to access secured or restricted areas (*e.g*., airports, ATM, datacenters). An iris recognition (IR) technique for identity authentication/verification is proposed in this research. Iris image pre-processing, which includes iris segmentation, normalization, and enhancement, is followed by feature extraction, and matching. First, the iris image is segmented using the Hough Transform technique. The Daugman’s rubber sheet model is the used to normalize the segmented iris area. Then, using enhancing techniques (such as histogram equalization), Gabor wavelets and Discrete Wavelets Transform should be used to precisely extract the prominent characteristics. A multiclass Support Vector Machine (SVM) is used to assess the similarity of the images. The suggested method is evaluated using the IITD iris dataset, which is one of the most often used iris datasets. The benefit of the suggested method is that it reduces the number of features in each image to only 88. Experiments revealed that the proposed method was capable of collecting a moderate quantity of useful features and outperformed other methods. Furthermore, the proposed method's recognition accuracy was found to be 98.92% on tested data.

## Introduction

When accessing secured sites such as ATMs, airports, and datacenters, individuals can prove their identities using standard security systems based on knowledge (*e.g*. passwords) or possession (*e.g*. tokens), but they are vulnerable to loss, theft, or guessing (Bhattacharyya et al., 2009). As a result, the researcher focuses his attention on biometrics. In recent years, biometrics has grown in prominence as a result of security concerns. Biometrics is one of scientific branches that uses computer technology to verify or identify individuals based on their physical and behavioral attributes, physiological traits that include the face, iris and retina (El-Sayed, Hassaballah & Abdel-Latif, 2016), as well as fingerprint and palm print, which are related to human’s body parts. Speech, gait, and keystroke are examples of behavioral characteristics that are related to an individual’s actions. Physiological characteristics are also more precise and provide more consistent findings (Jain, Ross & Prabhakar, 2004). Due to its unique composition, the iris is the most ideal physiological biometric characteristic for high security applications (Daugman, 1993; Bowyer, Hollingsworth & Flynn, 2008). This peculiar composition makes every iris unique. Even a person’s right iris is distinct from his or her left (Soliman et al., 2017). There is no genetic breakthrough in the iris makeup. The iris pattern is highly random and does not alter over a person’s lifetime (Muron & Pospisil, 2000). In recent years, iris recognition has proven to be one of the most reliable biometric technologies (Wildes, 1997; Daugman, 2009). Iris minutiae have a characteristic appearance. It’s sophisticated enough to be used as a signature (Daugman, 1994).

As a result, finding two persons with identical iris patterns is impossible (Boles & Boashash, 1998). As a consequence, the iris is commonly used in the identification process. Iris recognition techniques can achieve higher precision as compared to other biometric methods. In iris recognition, errors and rejects are uncommon, and the rate of error is the lowest of all the biometrics branches (Daugman, 1993). The iris recognition concept was first introduced in 1939 by Dr. Frank Burch, and it was used in 1990 for the first time when Dr. John Daugman developed its algorithms. Iris recognition as a biometric technique for identification has been a popular focus of research since this time. Iris recognition has been the subject of a number of researches, both for detection and verification, each with its own collection of benefits and disadvantages. Some of these studies had a high recognition rate but needed more storage due to the large number of extracted features, while others took longer to identify subjects. We focus on extracting only the most effective key features that can lead the system to correct classification in this paper. The first step in iris recognition is iris segmentation, which is followed by normalization and enhancement. To minimize the number of dimensions in which they are displayed, the segmented iris images are subsequently subjected to a feature extraction process. For this phase, the use of (DWT) and Gabor wavelet to extract the key features of the iris was recommended in order to improve the performance of the Iris Recognition System (IRS) and the storage of iris templates. SVM was eventually used to classify the process.

The major aim of this research work is to design an efficient biometric system based on various iris features to ensure precise identification of human. The limitations of traditional biometric system such as high false alarm rate are overcome by improving the accuracy of identification using deep learning techniques.

The following is how the paper is structured: In the second portion the Iris Structure is defined. The third portion introduces the “Relevant Literature”. The proposed approach is described in “Materials and Methods”. The findings of the experiments performed to test the proposed approach are found in “Results and Discussion”, and the conclusion is found in “Conclusions”.

## Iris Structure

The iris is a delicately colored component of the eye that is made up of several layers. Because of its pigmented properties, the epithelial layer makes the iris opaque. The pigment tissue, blood veins, and two iris muscles contract the pupil on the stromal surface (Kim, Kim & Kium, 2008).

**Figure 1:**
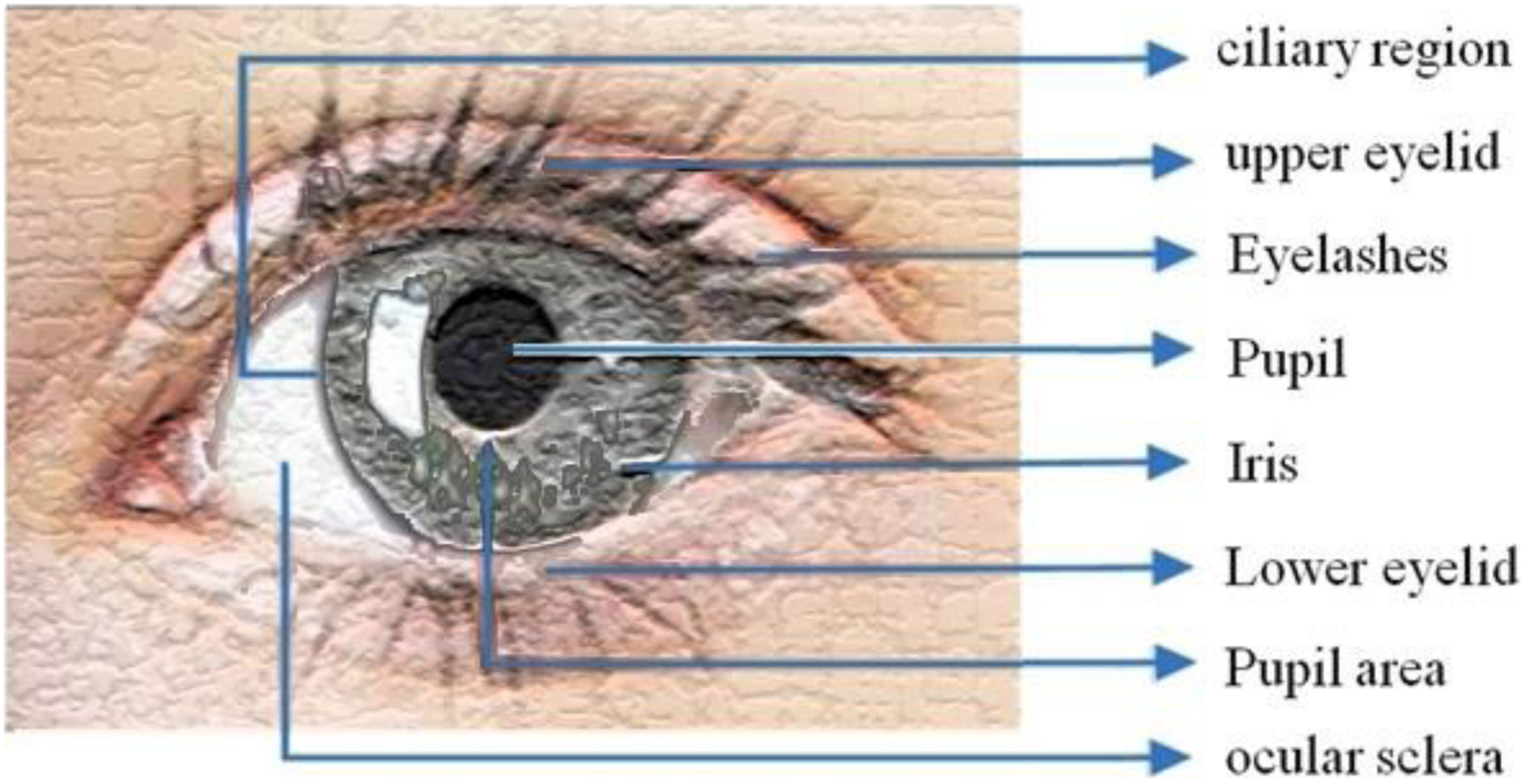
Iris structure.

The Collarette—which has a zigzag pattern—divides two zones: outer ciliary and inner pupillary (area) zones, Fig. 1 illustrates the eye image. Arching ligaments, ridges, furrows, crypts, rings, corona, freckles, and other distinguishing and distinctive features can all be found in the compound and multiplex arrangement. Iris characteristics are unpredictably distributed.

## Related Literature

Guo & Zheng (2018) suggested a gray projection algorithm for establishing identity authentication. They used improved Daugman and Point Hough Transform (PHT) algorithms to place the outer and inner boundaries, respectively. Following that, the iris image was normalized using line segment extraction, and the wavelet transform was used to remove the features. Finally, the Hamming Distance between two irises was measured in order to estimate the distance between two irises so that the iris could be recognized. The iris recognition system exhibited has a high level of robustness and is resistant to interference. The device has a high degree of accuracy, according to the trial results.

Meetei & Begum (2016) applied a BioHasing-based annullable iris biometric to iris biometric. The iris picture is segmented and normalized using the Hough transform and Daugman’s rubber sheet model. To create iris templates, minutiae are retrieved using DWT, ICA, and PCA. The iris template is converted using the suggested annullable iris biometric. In addition, the transformed iris templates are classified using SVM and RBF Kernels. Jagadeesh & Patil (2017) used a simple approach of segmentation that included a Gaussian filter followed by a canny edge detector following the pre-processing step. It makes it simple to locate the essential borders of the eye from the iris boundary. The Curvelets transform is used to extract features from segmented images so that the iris can be represented in significant dimensions. SVM was used to classify the data. Vyas et al. (2017) developed a unique iris recognition approach based on gray level co-occurrence matrices (GLCM) for feature extraction and a neural network as a classifier. The proposed technique maintained a high level of accuracy.

Kaudki & Bhurchandi (2018) offer a unique technique for iris detection based on fuzzy logic edge extraction and iris localization utilizing the Circular Hough Transform (CHT). Due to its computational ease, the Haar wavelet transform system is also utilized to extract the features. To compute template matching, the Hamming distance method is utilized. Archana et al. (2018) developed an open-source iris identification system to authenticate each iris’ identity and biometric performance. They used the Hough Transform for iris segmentation and the Gabor filter for feature extraction. Finally, the Hamming distance was employed to identify iris templates. Pattar (2019) proposed a breakthrough model as an enhancement of Chan-Vese technique by including Bspline approach for iris segmentation. To extract features in this experiment, the Local Binary Pattern (LBP) and GLCM processes were applied. Finally, SVM was utilized to determine whether or not the individual is approved. The proposed technique was evaluated using the NICE.I iris image database.

Entropy-based Occlusion Removal (Likitha, Gupta & Manikantan, 2015), proposed by Likitha, Gupta & Manikantan (2015), is a new technique for removing eyelashes that is utilized after iris localization. The Dual Tree Complex Wavelet Transform (DTCWT) is used in conjunction with the Discrete Cosine Transform (DCT) to extract shift-invariant features. Binary Particle Swarm Optimization (BPSO), which chooses the significant features extracted by DTCWT and DCT, is utilized for feature selection. The degree of similarity between the two photos is calculated using the Euclidean distance classifier.

Rana et al. (2019) proposed a technique that used Principal Component Analysis (PCA) and Discrete Wavelet Transformation (DWT) to identify key characteristics of an iris while reducing iris classification time. The pictures of iris were classified using SVM.

Deep learning based unified framework is proposed in Zhao & Kumar (2019) for accurate detection, recognition and segmentation of iris from raw eye images. The system architecture includes iris specific Mask R-CNN, normalization layer and feature extraction. This mask is used for iris segmentation. The training samples are along with its ground truth images. Normalization layer is used to perform iris and mask normalization before leaning the features of iris. After detecting iris, contrast enhancement process is proposed which is used to enhance the quality of the images. The features are extracted using fully connected layer of FeatNet. The features are learned by Extended Triplet Loss (ETL) function.

Qasmieh, Alquran & Alqudah (2021) proposed an accurate and fast IRS to address the noisy iris images specifically the noises come from eye occlusion and from specular reflection. It embraces a self-customized support SVM and CNN classification models. It used an optimized images processing techniques like iterative randomized Hough transform (IRHT) for iris region segmentation and used few significant features based on singular value decomposition (SVD) analysis for the classification. It used the Hamming distance to identify the subject’s unique iris pattern with high accuracy, security, and reliability.

Desoky, Ali & Abdel-Hamid (2012) proposed an IR algorithm in which a set of iris images of a given eye are fused to generate a final template using the most consistent feature data. Features consistency weight matrix is determined according to the noise level presented in the considered images. A new metric measure formula using Hamming distance is proposed.

Raffei, Dzulkifli & Rahman (2020) proposed a combination with neural network to improve the ability to match the iris features in mobile iris recognition system. The proposed method, a combination of Hamming distance and neural network has achieved a better result where it increases the existing method in term of accuracy for mobile iris recognition under non-cooperative environment.

Szymkowski et al. (2020) proposed retina diagnosing using biometric methods with SVM and clustering methods. The main aim of this research is to extract the features from retina images. First process is preprocessing which includes, denoising, contrast enhancement and normalization for improving image quality. Next process is vessel segmentation, binarization and thinning which is done by Gaussian matched filter with binarization using local entropy thresholding. Final process is minutiae extraction which is done by crossing number algorithm. After extract the minutiae the proposed method automatically counts the number of minutiae which helps to diagnose the disease. SVM is used to classify the detected eyes are healthy or unhealthy.

Li et al. (2021), authors proposed a segmentation method for precise segmentation of iris images by incorporating deep learning model. The importance of proper segmentation of iris for accurate recognition was addressed. The reasons for noises in the input image such as low resolution, light reflections, blur, motion and occlusions affecting the accuracy of segmentation was analyzed. The interleaved residual U-Net (IRU-Net) was implemented to construct the outer boundary and inner boundary of iris based on salient features. The scanning and clustering of salient features was performed to construct the external boundary whereas the inner boundary was constructed by intersecting the salient features obtained from the deep learning model.

## Materials and Methods

**Figure 2:**
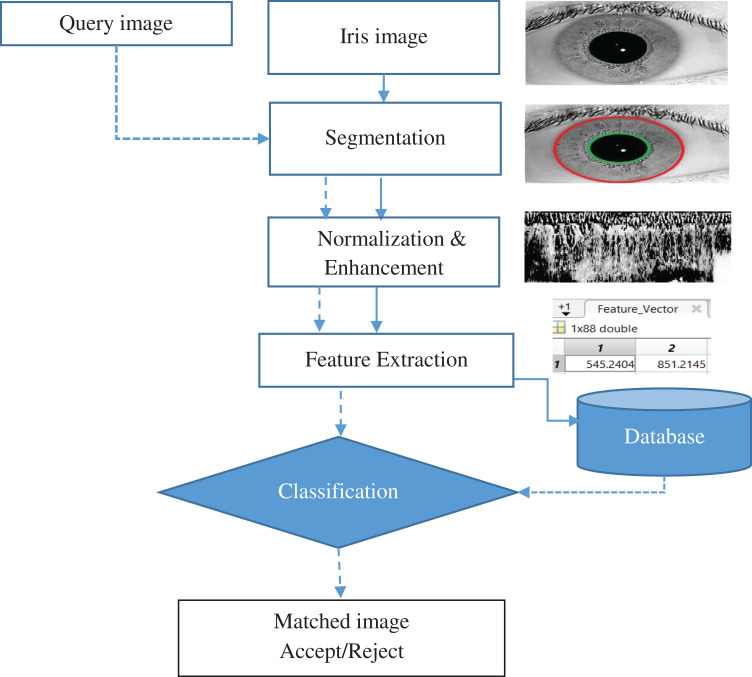
Iris recognition system.

In general, there are four processes to iris recognition: (1) Acquisition of the iris Image; (2) pre-treatment (Segmentation, normalization and Enhancement): (3) extraction of features: (4) then there's the process of matching. The suggested approach can be explained as exhibited in Fig. 2, in which an iris template is formed by performing the required steps of iris recognition and then compared to the templates stored in the iris database. The segmented iris area is normalized to a rectangular form with firm polar dimensions using Daugman’s rubber sheet model. The characteristics were extracted using a Gabor Wavelet and DWT combination. For classification, a multiclass SVM was utilized.

### Iris image capture

The acquisition of an image of the iris is the initial stage in the iris recognition process. The person’s iris is photographed in a number of high-quality photographs. The photos should, however, clearly show the complete eye, especially the iris and pupil. The procedure of noise reduction is avoided with a good and clear image, as well as faults in computing. An infrared sensor or a high-resolution digital camera can be utilized for this purpose. The IIT Delhi iris image database was used to complete the tasks listed above.

### Iris image pre-processing

An eyelid, eyelash, pupil, and other irrelevant components of the acquired image. They should be removed. The three steps in this level are iris localization/segmentation, normalization, and enhancement.

#### Iris segmentation/localization

Either the acquired image of an eye or just the iris can be used for recognition. The problem is that the eye may have a lot of redundancy, necessitating the consideration of a high number of features for recognition. Another disadvantage is that it leads to the feature picker selecting features that aren’t required for recognition. Iris segmentation was used to segment the iris portion of the ocular image. The iris area is bounded by two circles, one for the sclera/iris (outer boundary) and the other for the pupil/iris (inner boundary). As a result, only the iris, a particularly rich feature of the eye, is obtained *via* segmentation. Various segmentation techniques are used, including Daugman’s. The integrodifferential operator was used to detect the pupil and iris boundaries in the iris area (Daugman, 1993). For determining the edges that correspond to the iris boundaries, Wildes (1997) used the 1^st^ derivative of picture intensity. While (Ma et al., 2004) used the Hough transform to detect the pupil and iris borders.

### Hough transformation

In this study, the Hough Transformation was used to determine the iris area. This technique was used to define the parameters of geometric shapes in an image, such as circles and lines. The radius, center, and pupil area of the iris may all be calculated using the circular Hough transform. The Hough algorithm creates an edge map by computing the first derivatives of intensity values or gradients in an eye image. The surrounding points on the circle at various radii are taken for each edge pixel in the edge map, and votes are cast to acquire the maximum values that create the parameters of circles in the Hough space. The parameters *x_c_*, *y_c_* which are the center coordinates and the radius ‘*r*’ can be found through the equation: $x_c^2 + y_c^2 - {r^2} = 0$. The maximum point corresponds to the center coordinates ( ${x_c}$, ${y_c}$) in the Hough space, and the radius of the circle can be given by the edge points. We used vertical gradients to determine the outer boundary (iris-sclera) and to reduce the impact of the eyelids, which were horizontally oriented to determine the iris boundary and vertical gradients to limit the impact of the eyelids. All of the pixels on the edges are altered when a circular Hough Transformation is applied. In the Hough space, there are fewer edge points from which to cast votes, which not only improves circle localization accuracy but also efficiency (Rana et al., 2019).

#### Iris normalization

**Figure 3:**
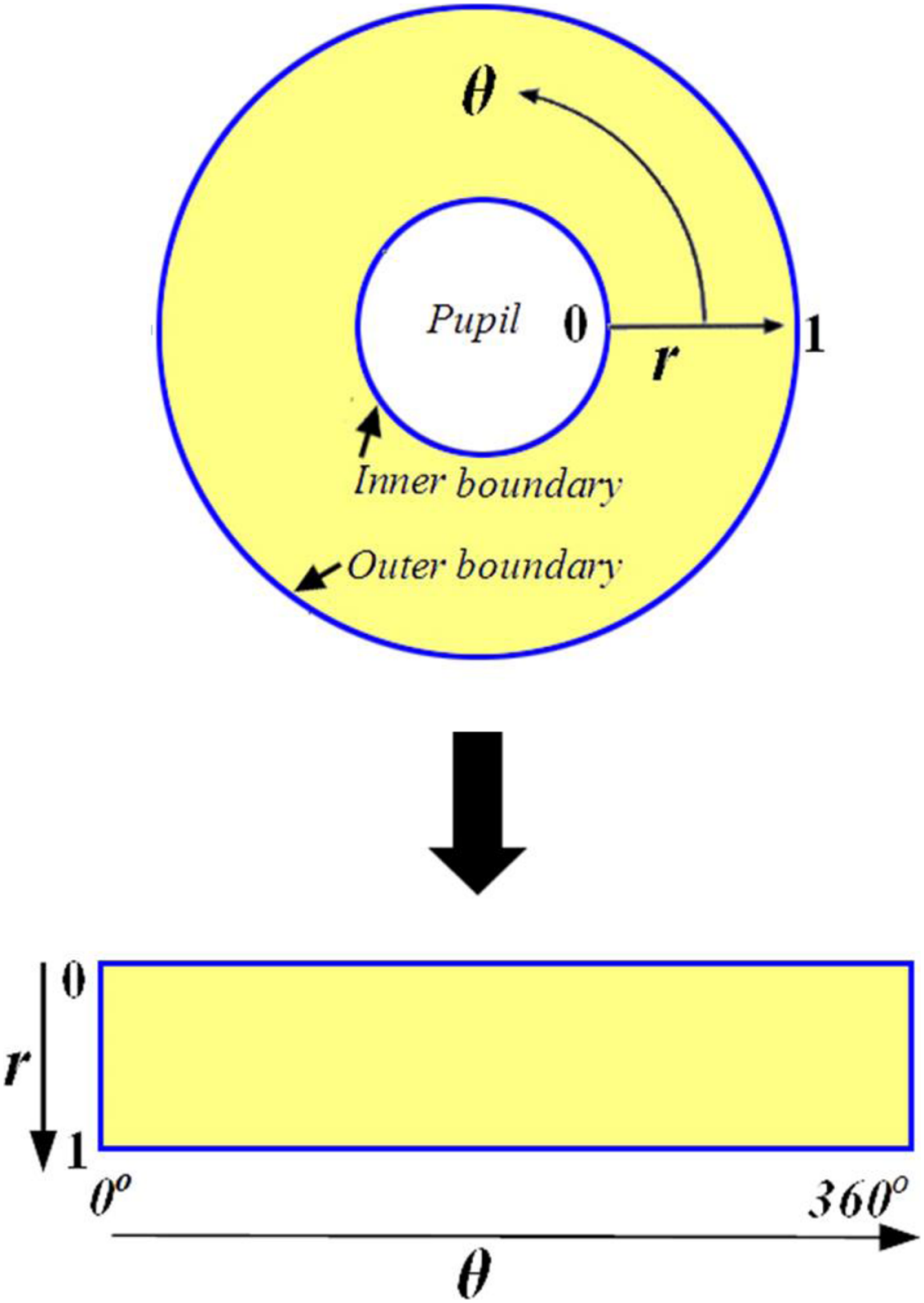
Daugman rubber sheet model.

The normalization procedure produces iris areas with constant size after properly segmenting the iris area. This technique can be carried out using the Daugman rubber sheet model (Daugman, 1994; Ma et al., 2004; Podder et al., 2015). The reference point is the pupil center, and radial vectors run across the iris area, as illustrated in Fig. 3. Angular resolution refers to the radial lines that circle the iris area. Because the pupil is not concentric with the iris, rearranging points based on the orientation around the circle necessitates the use of a basic formula. The grey values of these pixels identify the entire iris area. This information’s can be determined by coordinate’s combines of inner and outer boundaries. This model rescales each point inside the iris region to a pair of polar coordinates (*r*, θ) where ‘*r*’ lies in the interval [0, 1] and ‘θ’ is an angle in the range of ‘0’ to ‘360’ (2 $\pi$) degree.

So. If *s*(*x*, *y*) is an iris image represented in Cartesian coordinates and *s*(*r*, θ) is the representation in polar coordinate. And if (*x_i_*, *y_i_*) and (*x_o_*, *y_o_*) is the inner boundary and outer boundary unit in Cartesian coordinates respectively, then

(1) $$s\left( {x\left( {r,\theta } \right),y(r,\theta )} \right) \to f(r,\theta )$$

(2) $$x\left( {r,\theta } \right) = \left( {1 - r} \right){x_i}\left( \theta \right) + r{x_o}(\theta )$$

(3) $$y\left( {r,\theta } \right) = \left( {1 - r} \right){y_i}\left( \theta \right) + r{y_o}(\theta )$$

In the above equation, $r = {P \over {M + 1}}$, where $p = 1,2, \ldots M$ and $\theta = {q \over N}$, $q = 1,2, \ldots N$.

The sample rate, as well as the angle and radial direction, are represented by *M* and *N*, respectively. The following is a description of the normalizing algorithm:

Step 1: Based on iris boundary localization of iris image $s\left( {x,y} \right)$, calculate the parameters of $({x_i},{y_i},{r_i})$ and $({x_0},{y_0},{r_0})$. Where the subscript *i* and *o* means the inner and outer boundary.
Step 2: The following formula can be used to calculate the distance between the pupil’s center and the iris’s center: $r\prime = \sqrt {{{({x_i} - {x_o})}^2} + {{({y_i} - {y_o})}^2}}$
Step 3: Connection direction angle is also calculated. $\varphi = arc\tan \left({{{y_i} - {y_o}} \over {{x_i} - {x_o}}}\right)$
Step 4: Choose the pupil’s center as your pole. In the polar system $r(\theta ) = {r_p}$. For the outer boundary of the iris, $\theta = q{\pi \over {180}}$, where $j = 1,2, \ldots N,R\left( \theta \right) = r^\prime \cos \left( {\pi - \theta - \varphi } \right) + \sqrt{r_o^2 - r^{\prime2} + (r^\prime \cos (\pi - \theta - \varphi )^2)}$
Step 5: The grey values of the normalizing iris for each pixel can be calculated using those grey $\left( {x,y} \right)$ coordinates and the following equations:

(4) $${R_p} = \left(1 - {p \over {M + 1}}\right) * r\left( \theta \right) + {p \over {M + 1}}R(\theta )$$

(5) $$x = {X_p} + {R_p}cos\theta ,y = {Y_p} + {R_p}sin\theta$$

#### Enhancement

**Figure 4:**
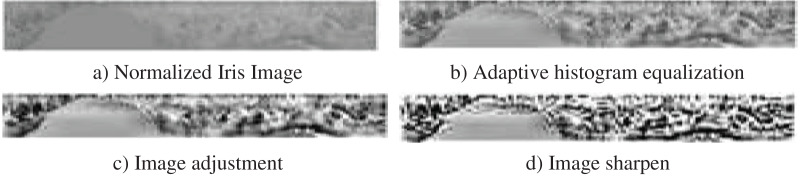
Enhancement steps.

Depending on the application, enhancement is a technique that is used to increase the image quality. In iris recognition, the low contrast image of a segmented iris must be corrected. As a result, Adaptive Histogram Equalization, Image Adjustment and Image Sharpen are applied in that order. Photographs at various levels are depicted in Fig. 4. It is observed from that figure how features in image is enhanced in each stage of preprocessing.

### Feature extraction/encoding

The feature extraction procedure is a type of dimension reduction that is unique. Feature extraction is the process of converting the input data into a set of features. When the features are carefully selected, the features collection is anticipated to extract the critical information from the input data in order to complete the required procedure using this reduced representation rather than the full-size input. Analyze the Principal Components (PCA), Kernel PCA, Linear Discriminate Analysis (LDA), and SVM are examples of prominent pixel-based feature extraction algorithms that produce good results. The fundamental drawback of these methods is that features are inversely proportional to speed and proportionate to accuracy. It means that as the number of selected features increases, the probability of accuracy increases as well, although the rate of speed decreases. It took extra time to calculate additional features. Wavelets devised a third method for identifying the best solution to such issues (Sheikh & Thakare, 2016). The segmented iris portion is used to extract various features. They are as follows: **Gabor Features**. This is a linear filter that detects edges. This filter representation is similar to that of the human visual system, and it has the ability to reflect texture and discriminate it successfully. As a result, these features are used in this research. **Wavelet Features.** Wavelet transform is a sub sampling technique that separates an image into several sub bands. Because the image is separated into sub bands, this altered version of the image can extract an n-number of characteristics. Image features such as mean, standard deviation, low band, and high band are called features, and they are merged to generate the ultimate feature vector (Tallapragada & Rajan, 2010). Finally, all of the retrieved features are merged and formed into a high-dimensional feature vector. The purpose of creating such a large feature vector is to improve classification accuracy.

#### Wavelet transform

The Wavelet Transform will exemplify the signal in terms of time and frequency. It may clarify important aspects of a signal, such as trends, breakdown points, higher derivative discontinuities, and self-similarity (Misiti et al., 2001). The major benefit of the wavelet transform is its local and multi-resolution feature extraction capability. The wavelet transforms often called a ‘mathematical microscope’. The ways by which wavelet transform can be developed are Continuous wavelets transform (CWT) and discrete wavelet transform (DWT).

**Figure 5:**
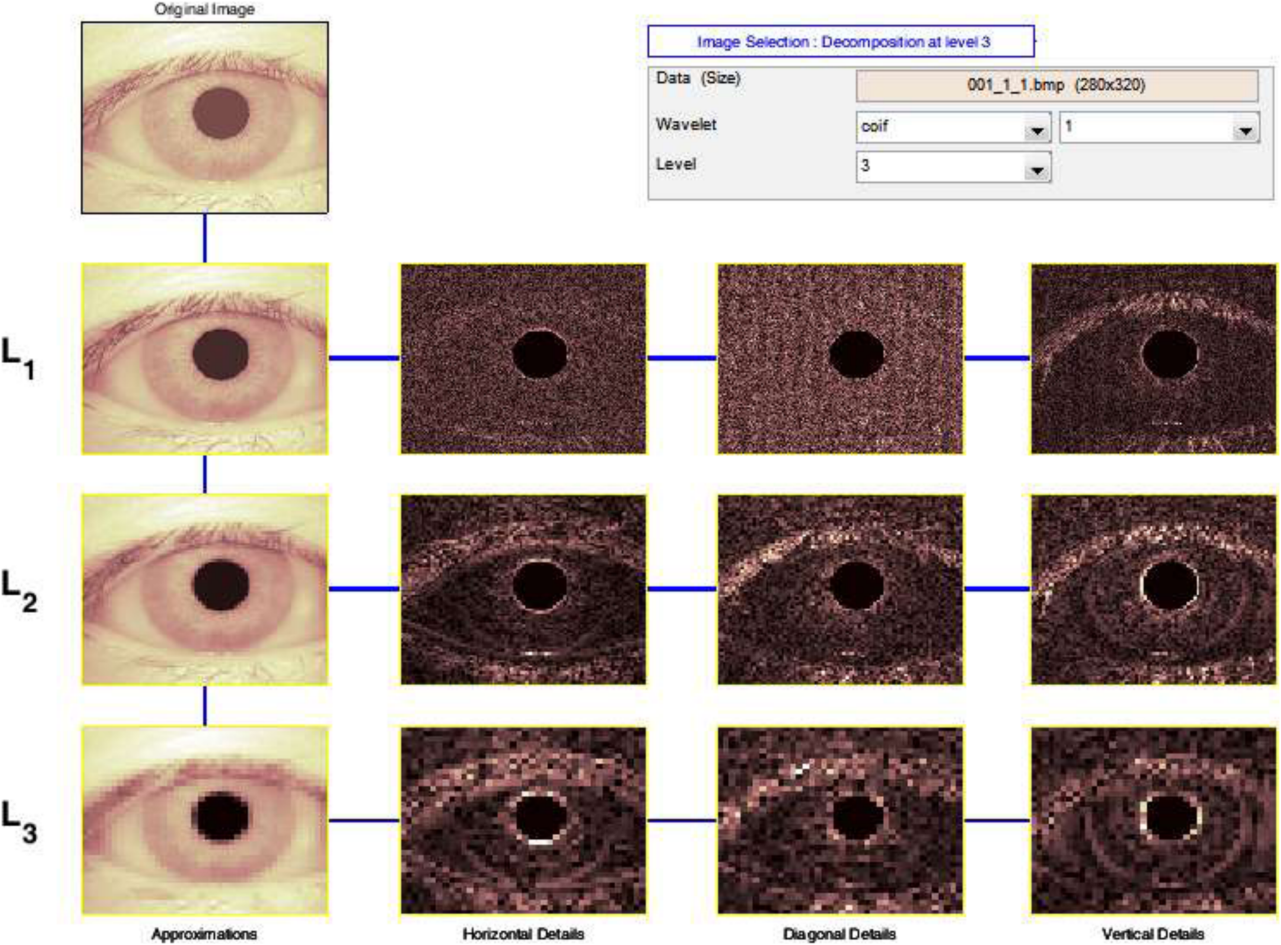
Output of 3-levels tree of wavelet decomposition.

The Discrete Wavelet Transform (DWT) is a rapid Wavelet Transform calculation method based on sub-band coding. It is simple to use and saves time and resources while performing calculations. When techniques for decomposing discrete-time signals were devised in 1976, DWT was founded (Birgale & Kokare, 2009). As shown in Fig. 5, the DWT is performed by filtering the discrete time-domain signal with successive low-pass and high-pass filters.

When more precise low-frequency information and compact regions are required, wavelet analysis allows the use of long-time intervals. It takes a lot of time and effort to compute wavelet coefficients at all feasible sizes, and it generates a lot of data. As a result, our calculations are limited to a subset of scales and places. It turns out that if we choose scales and positions based on the powers of two so-called dyadic scales and positions, our analysis will be significantly more efficient and accurate. The DWT provided by provides us with such an analysis Eq. (6).

(6) $$DWT = \sum\limits_{k = 1}^\infty {\sum\limits_{l = - \infty }^{ + \infty } q } \left( {k,l} \right)\psi ({2^{ - k}}t - l)$$

Filters were introduced in 1988 as an effective approach to implement this method. This approach is known in the signal processing field as a two-channel sub-band coder. This is a helpful filtering approach that constructs a box through which a signal flows and wavelet coefficients emerge fast using a simple wavelet transform, let’s say that

(7) $$\emptyset (x) = \sum\limits_n {{h_\emptyset }} \left( n \right)\sqrt 2 \emptyset (2x - n)\ {\rm and}$$

(8) $$\psi (x) = \sum\limits_n {{h_\psi }} \left( n \right)\sqrt 2 \psi (2x - n)$$

$\emptyset (x)$ and $\psi \left( x \right)$ can be expressed as linear combinations of double-resolution copies of themselves. Here ${h_\emptyset }$ in (7) and ${h_\psi }$ in (8) the expansion coefficients are called scaling and wavelet vectors, respectively. They are the filter coefficients of fast wavelet transform (FWT), ${W_\emptyset }\left( {j,m,n} \right)$ Approximate coefficients, $W_\psi ^H\left( {j,m,n} \right)$ Horizontal coefficients, $W_\psi ^V\left( {j,m,n} \right)$ Vertical coefficients and $W_\psi ^D\left( {j,m,n} \right)$ Diagonal coefficients. Here ${W_\emptyset }\left( {j,m,n} \right)$ the original image whose DWT is to be computed (Birgale & Kokare, 2009).

#### Gabor wavelet

Gabor wavelets (filters) have frequency and orientation representations that are akin to those of the human visual system, and they were created with texture representation and discrimination. Pattern analysis applications make extensive use of Gabor filters (Shen, Bai & Fairhurst, 2007; Liu & Wechsler, 2002; Meshgini, Aghagolzadeh & Seyedarabi, 2013). Another notable advantage of Gabor filters is their invariance to lighting, rotation, size, and translation. Photometric disturbances like light variations and picture noise are also tolerated. In the spatial domain, a two-dimensional Gabor filter is defined as a Gaussian kernel function modulated by a complex sinusoidal plane wave, as shown:

(9) $$G\left( {x,y;\lambda ,\theta ,\psi ,\sigma ,\gamma } \right) = {{{\lambda ^2}} \over {\pi \gamma }}\exp \left( {{{ - 1} \over {2{\sigma ^2}}}\left( {{x^{\prime 2}} + {\gamma ^2}{{y^{\prime 2}}}} \right)} \right)\exp\left( {i\left( {{{2\pi x^\prime} \over \lambda } + \psi } \right)} \right)$$

where,

(10) $$x\prime = x\cos \left( \theta \right) + y\sin \left( \theta \right),\matrix{\ {y\prime = - x\sin \left( \theta \right) + y\cos \left( \theta \right)} \hfill \cr }$$

where $\lambda$ is the wavelength of the sinusoidal factor, $\theta$ represents the orientation of the normal to the parallel stripes of a Gabor function, $\psi$ is the phase offset, $\sigma$ is the standard deviation of the Gaussian envelope and $\gamma$ is the spatial aspect ratio which specifies the ellipticity of the Gabor function’s support. Log Gabor filters (Shen, Bai & Fairhurst, 2007; Kumar & Rao, 2014) are used in the proposed algorithm, and their frequency response is as follows:

(11) $$G\left( f \right) = exp\left( {{{ - {{(log(f/{f_o}))}^2}} \over {2{{(log(\sigma /{f_o}))}^2}}}} \right)$$

where ${f_o}$ represents the center frequency, and $\sigma$ gives the bandwidth of the filter.

#### Generating features

1. Practically, the function waveletTransform utilities a wavelet transform and dwt2 function in matlab form and generates 40 features. An image inputs to process and extract wavelet coefficients. The output is 1 × 20 feature vector containing the first 2 moments of wavelet coefficients (mean and standard deviation coefficients).
2. The following Function for computing Gabor features of a gray-scale image This function calculates Gabor features and generates 48 features, 24 features generated by Mean Amplitude for each scale and orientation is returned, and 24 features generated by Square Energy. Mean-squared energy. There are potentially many arguments, here is all parameters have defaults and the usage can be as simple as gaborWavelet(im); For maximum speed the input image should have dimensions that correspond to powers of 2, but the code will operate on images of arbitrary size.

Filters are constructed in terms of two components. The radial component, which controls the frequency band that the filter responds to. The angular component, which controls the orientation that the filter responds to. The two components are multiplied together to construct the overall filter. Construct the radial filter components. First construct a low-pass filter that is as large as possible, yet falls away to zero at the boundaries. All log Gabor filters are multiplied by this to ensure no extra frequencies at the ‘corners’ of the FFT are incorporated as this seems to upset the normalization process when calculating phase congruency. For each point in the filter matrix calculate the angular distance from the specified filter orientation. To overcome the angular wrap-around problem sine difference and cosine difference values are first computed and then the atan2 function is used to determine angular distance.

### Classification

The aim of classifying two iris templates generated during the feature extraction stage is to determine their similarity. When comparing the same iris templates, this method provides one set of values, while when comparing iris templates from different people’s eyes, this method gives another set of values (Rana, Azam & Akhtar, 2017). The conclusion of this training phase will determine whether or not the two iris models belong to the same person. The SVM is one of the most widely used machine learning methods for classification and regression applications. As a result, this study recommended the classification of iris images. Its goal is to develop a function that can predict which class the new and old points belong to with accuracy. The Support Vector Machine is basically a binary classifier that efficiently distinguishes between the two types of data. It is the most promising method and technique in comparison to other approaches and techniques. SVM scales effectively to high-dimensional data, and the tradeoff between classifier complexity and error may be precisely managed.

SVMs and kernel methods also have the advantage of being able to develop and apply a kernel for a specific task that can be applied directly to the data regardless of the feature extraction step. It's critical in situations where the feature extraction method destroys a significant amount of the data's structure (*i.e*. text processing). Binary Classification and Multiclass Classification are the two modes in which SVM can be used. The fundamental goal of SVM in a binary classification problem is to partition the data in the best way possible. When we need to classify two data sets, such as iris identification, we utilize binary classification (matched or unmatched). SVM was basically developed for two-class classification problems in mind.

Support Vector Machine can deal with two cases. 1) When the data are linearly separable and b) the data are nonlinearly separable. A linear decision’s many boundaries will split the data into linearly separable data. Only one of these, though, achieves maximum division. The major goal is to classify it to one group of datasets relative to others using a decision boundary, which we don’t want to happen, thus a maximum margin classifier or hyperplane as a visible solution comes into play. The data points nearest to the decision surface are called support vectors. The data points that the margins push up against are known as Support Vectors. They are the most difficult to categorize. The main challenge here is to find the only ideal margin of the separating hyper plane $w.{x^T} + b = 0$, the one that gives the most margin between the classes. This margin guarantees that the rate of misclassification is kept to a minimum. Margin also has the benefit of avoiding local minima and allowing for improved classification (Auria & Moro, 2008).

To translate this problem into a formula. Assume that *x* is a set of input feature vectors and *y* is the class label. The input feature vectors and the class label can be represented as $\{ {x_i},{y_i}\}$, where *i* = 1, 2,…,*N* and *y* = {−1,1}. The following is a representation of the separating hyper plane:

(12) $$w.x + b = 0$$

which implies,

(13) $${y_i}(w.{x_i} + b) \ge 1;i = 1,2 \ldots N$$

(*w*, *b*) can take on a variety of different values, resulting in a separate hyper plane. It is thought that points frequently lie between two data classes in such a way that a margin exists between them. By treating it as a quadratic issue, SVM optimizes this margin (Auria & Moro, 2008; Barpanda et al., 2018). So, the SVM for a separable case is:

(14) $$\min{1 \over 2}{\left\| w \right\|^2}$$

(15) $${y_i}(w.{x_i} + b) \ge 1; \forall {x_i}$$

The cost parameter C in SVM models determines the tradeoff between tolerating training errors and imposing strict margins, allowing some flexibility in separating decision borders. It produces a squishy border that allows for certain misclassifications. Optimization problem takes the form

(16) $$\min {1 \over 2}{\left\| {\rm{w}} \right\|^2}+{\rm C} \sum\limits_{{\rm{i}} = 1}^{\rm{N}} {\xi}_{\rm{n}}$$

where ${\xi}_i>1$ means that sample *i* is misclassified and ${y_i}(w.{x_i} + b) \ge 1 -{\xi_i}$, C trades-off margin width and misclassifications.

As previously said, SVMs are designed to cope with binary classification, however in today’s environment, we also have a lot of data to categorize. Amounts or a trace of a variable’s values across time, such as a year, month, week, and so on, are reflected in time series data. There will be more than two classes in this. As a result, multiclass classification is required. The term “multiclass classification” refers to when there are more than two classes that need to be distinguished. There are several types of multiclass SVM, but the most widely used are one-*vs*-all (OVA), in which each class is compared to all other classes as if they were one (Bottou et al. 1994), *i.e*., one classifier per class; and one-*vs*.-one (OVO), in which each class is compared to all other classes individually (Knerr, Personnaz & Dreyfus, 1990), *i.e*., one classifier per pair of classes. The one-*vs*.-all strategy is useful since it is easy to understand. This is the most popular method, and it’s a good starting point. With n samples, a one-*vs*.-one classifier fails to scale. The general case of multiclass SVM algorithm for the separable case after adding slack variables to the optimization problems can be given as follows:

(17) $${\min _{{w_1},{w_2}, \ldots ,{w_k}}}{1 \over 2}\sum\limits_k {w_k^T} {w_k} + C\sum\limits_{({x_{i,}}{y_{i,}}) \in D} {{\xi _i}}$$

${\rm{w}}_{{{\rm{y}}_{\rm{i}}}}^{\rm{T}}{{\rm{w}}_{\rm{k}}} - {\rm{w}}_{\rm{k}}^{\rm{T}} \ge 1- \xi i, ({x_i}, {y_{i,}}) \in D, k \in \left\{ {1,2, \ldots ,{\rm{K}}} \right\},{\rm{k}} \ne {y_{i,}}, \xi_{i} \ge 0$

The fundamental purpose of SVM (Auria & Moro, 2008) is to isolate data by decision boundary and use the kernel method to expand it to non-linear boundaries. Because SVM is multifunctional, different Kernel functions can be assigned to the decision function. Among them are the radial basis function (RBF), linear, polynomial, and sigmoid functions. The RBF is the most widely utilized kernel type in SVM. This is because their responses are localized and finite across the entire range of the real *x*-axis. As a result, it made use of this work. The RBF kernel is defined as follows for two samples *x* and *x*′ that have been represented as feature vectors in some input:

(18) $$K\left( {x,x^\prime} \right) = {e^{\left( - {{{{\left\| {x - x^\prime} \right\|}^2}} \over {2{\sigma ^2}}}\right)}}$$

${\left\| {x - x\prime} \right\|^2}$, may be recognized as the squared Euclidean distance between the two feature vectors. σ is free parameter. An equivalent definition involves a parameter *γ* = ${\textstyle{{1} \over {2 \sigma^2}}}$:

(19) $$K\left( {x,x\prime} \right) = {e^{( - \gamma {{\left\| {x - x\prime} \right\|}^2})}}$$

During iris recognition, the SVM can make one of two decisions: accept or reject a person.

## Results and Discussion

### Images dataset

**Figure 6:**
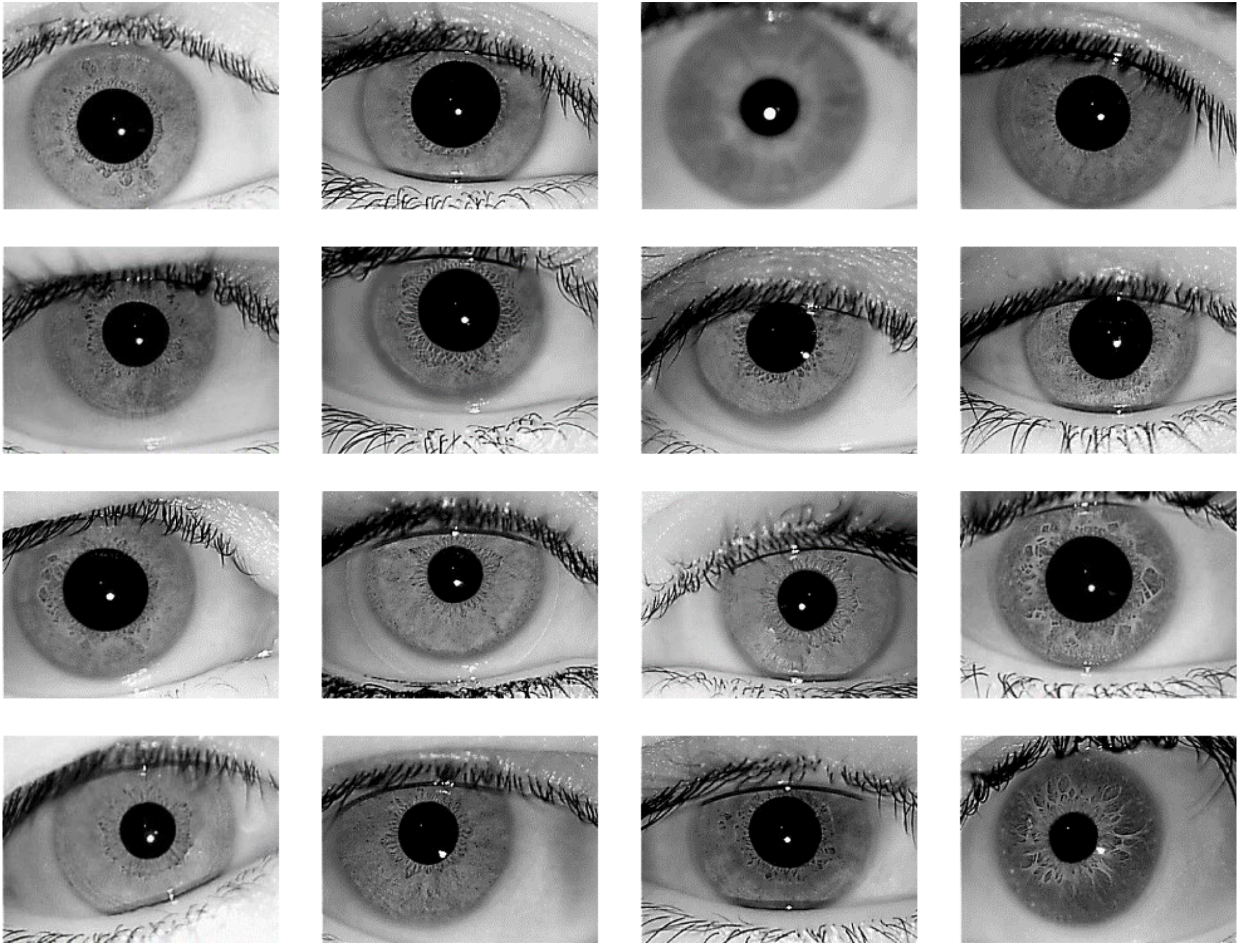
16-Sample images from IITD Iris dataset.

The proposed iris recognition system is evaluated in this paper using one of the most widely available datasets: the IITD V1.0 dataset (Kumar & Passi, 2010; https://www4.comp.polyu.edu.hk/~csajaykr/IITD/Database_Iris.htm) which contains iris photos from 224 classes (users). The bitmap (*.bmp) format is used for all of the images. All of the subjects in the dataset are between the ages of 14 and 55, with 176 men and 48 girls. The database contains 1120 photos with a resolution of 320 × 240 pixels. As a result, there are 224 classes in this dataset, each having five sample iris photos, and all of these images were taken indoors using the near-infrared (NIR) wavelength. Figure 6 shows 16-sample images from IITD Iris dataset.

All experiments are conducted on a Windows-based machine with Intel (R) Core (TM) i7-7500U CPU@2.70 GHz processor, 16 GB RAM, and MATLAB R2017a installed on it. In the verification mode, the proposed system's performance is assessed. In this mode, each iris image is examined as a single test image, with all others balanced. Table 1 shows the relationship between recognition rate and various training/testing ratios. Recognition Rate (RR), Obtained for IITD databases by applying the proposed technique, along with other pre-processors for different training: testing ratios. The higher the training ratio, the higher the accuracy of recognition.

**Table 1:** Recognition rate of Gabor wavelet, DWT and proposed method using IITD iris dataset.

Technique	No. of features	Training %	Testing %	RR %
Gabor wavelet	48	20	80	57.232143
		40	60	74.553571
		60	40	86.160714
		80	20	91.339286
DWT	40	20	80	67.053571
		40	60	83.571429
		60	40	90.803571
		80	20	95.892857
Proposed method	88	20	80	75.892857
		40	60	90.089286
		60	40	95.714286
		80	20	98.928571

The table also shows the number of features derived using the Gabor wavelet and the Discrete Wavelet Transform. On the basis of the accuracy rate, Table 2 contrasts the proposed system to the other current methods. The proposed method’s efficiency in the verification mode is assessed. Each iris image is treated as a single test image that matches all other images in the database in this mode. The association between the recognition rate and various training and testing ratios is shown in Table 1. The more training ratio, the more recognition rate accuracy. Moreover, the table shows the number of features extracted using Gabor wavelet and Discrete Wavelet Transform. Table 2 shows a comparison between the proposed method and the existing methods based on the accuracy rate, using the same dataset (IITD iris dataset).

**Table 2:** Accuracy comparison of our proposed technique with existing methods.

Method	(Mrinalini et al., 2015)	(Vyas et al., 2017)	(Ali, Suandi & Abdullah, 2018)	(Vyas, Kanumuri & Sheoran, 2016)	(Qasmieh, Alquran & Alqudah, 2021)	Proposed method
Accuracy (%)	94.04	97.83	96.5	96.33	97.12	98.92

## Conclusions

A new iris recognition method is introduced in this paper. For the segmentation job, the device used the Hough Transform process, with the Daugman rubber sheet model for normalization, Gabor wavelets, and wavelet transforms for feature extraction. Finally, multiclass SVM was used with caution to complete the matching job. The proposed approach uses only 1 × 88 vectors to create an iris signature. The speed can be improved by using a signature of this length. The developed approach in this study reduced the number of features while maintaining a high degree of accuracy. The proposed technique’s accuracy is 98.92%, which is higher than some other current approaches for accurate comparison.

## Additional Information and Declarations

### Data Availability

The following information was supplied regarding data availability: The data is available at figshare: Abdellatif, Mohammed (2022): Paper Verification_IITD Iris - DWT. figshare. Software. https://doi.org/10.6084/m9.figshare.19199441.v1. Abdellatif, Mohammed (2022): Paper Verification_IITD Iris - GW. figshare. Software. https://doi.org/10.6084/m9.figshare.19199456.v1. The IIT Delhi Iris Database (Version 1.0) is available at https://www4.comp.polyu.edu.hk/~csajaykr/IITD/Database_Iris.htm.
